# On Folk Devils, Moral Panics and New Wave Public Health

**DOI:** 10.15171/ijhpm.2019.78

**Published:** 2019-09-29

**Authors:** Russell Mannion, Neil Small

**Affiliations:** ^1^Health Services Management Centre, University of Birmingham, Birmingham, UK.; ^2^Faculty of Health Studies, University of Bradford, Bradford, UK.

**Keywords:** Moral Panic, Obesity, Public Health Policy, Medical Sociology

## Abstract

New wave public health places an emphasis on exhorting individuals to engage in healthy behaviour with good health being a signifier of virtuous moral standing, whereas poor health is often associated with personal moral failings. In effect, the medical is increasingly being collapsed into the moral. This approach is consistent with other aspects of contemporary neoliberal governance, but it fuels moral panics and creates folk devils. We explore the implications and dysfunctional consequences of this new wave of public health policy in the context of the latest moral panic around obesity.

## The Medical and the Moral

Since it emerged as an organised endeavour across western societies in the 19th century, public health has shifted focus a number of times. Initially it represented a collective response to advances in scientific knowledge about the sources and spread of infectious diseases, then the predominant cause of death. The emphasis was on large scale public programmes to improve hygiene, sanitation and air quality. While the pioneer sanitary reformers were motivated by a wish to reduce infectious disease and to harness the advances of science they were also motivated by a strong moral commitment, inspired for many by a Christian ethos, to promote the well-being of the populous.^1^ Deaths from infectious diseases in England and Wales dropped from 25.9% of all deaths in 1911 to 0.7% of all deaths in 2013.^2^ The predominant cause of death by the mid-20th century was non-communicable disease. In 1971, 50% of all deaths were from heart disease, this rate dropped to around 28% by 2013.^2^ This drop can be attributed to improvements in treatment and also to a focus on addressing causes of death, including via a smoking ban in all enclosed work spaces which was introduced in England in 2007. Deaths from cancer have risen throughout the 20th century, 6% in 1911, 29% in 2013, but these deaths characteristically now occur later in life. This shift in the patterns of death has occurred at the same time as increasing life-expectancy.^2^

This changing pattern of disease is reflected in a shift in public health policy. During the last decades of the 20th century the focus moved to one that sought to combat physical inactivity, smoking, alcohol consumption and poor diet. These were seen as modifiable risk factors, if these risk factors could be reduced there would be less non-communicable disease later in life. In the 21st century, while the focus has remained on addressing things that are seen as causal factors of non-communicable disease, the emphasis has shifted towards programmes which seek to promote personal responsibility, with individuals required to exercise self-discipline in the areas health professionals define as high risk. Individuals are exhorted to engage in healthy behaviour with good health being a signifier of virtuous moral standing, whereas poor health is associated with personal moral failings.^3^

This shift from a collective moral commitment, evidenced in public works programmes, via a combination of legal and behavioural change to a concern with the moral failings of those who are seen as putting their health at risk has occurred at the same time as a shift in the role of the state in healthcare, within a broader shift in the nature of government. These are shifts that have been well-documented within the neoliberal governmentality literature,^4,5^ with recent extensions exploring the role of pastoral power in promoting adherent patient subjectivities.^6^ According to Foucault^7^ the logic of neoliberal governmentality is to “to extend the rationality of the market, the schemes of analysis it proposes, and the decision-making criteria it suggests to areas that are not exclusively or not primarily economic” (p. 79). It identifies dispersed mechanisms and technologies of power. Some power is still “old fashioned” sovereign power of command, in public health this might be banning or taxing something or offering inducements to encourage desired actions. But some power is now disciplinary power and this is concerned with the formation of motives, desires and character in individuals. The effect it seeks is that “disciplined” individuals acquire the habits, capacities and skills that allow them to act in socially appropriate ways without the need for the exercise of external coercive power.^8^ But those individuals who do not act in a disciplined way find that their subsequent encounters with health services involve their being given communicative messages at whose core is social and moral opprobrium for making poor lifestyle choices. Those that make poor choices are stigmatised and a moral distance is created between the well-behaved and the miscreants who are behaving badly.

In effect the medical is increasingly being collapsed into the moral.^9^ There are three main consequences of this. The first is one of “dissolving the bonds of social solidarity”^10^ as a denuded sense of citizenship is created in those subjected to moral distancing.^11,12^ The second concerns the way moral distance allows the state to step aside from what had previously been seen as part of its welfare responsibilities. A new quasi-state welfare apparatus emerges which is designed to outsource welfare and, in so doing, reduce the welfare bill. The third consequence is that a shift to the moral does not work, it does not take cognizance of biological, structural and social causation and it misreads what motivates choice in individuals. We will explore this analytic weakness in relation to obesity below.

If a moral distance from those seen to not be accepting personal responsibility for their behavior is created the next step is to portray those so distanced as a risk not just to themselves but to others. That risk is not the contagion, and the need for segregation to prevent the spread of disease from the few to the many, that the early public health exponents were concerned with but a risk to the economic well-being of the favored citizenry, those whose behavior is seen as appropriately disciplined.

Beck^13^ has argued that there is a new form of modernity which he terms “risk society.” Such a society has a systematic way of dealing with hazards and insecurities induced and introduced by modernization. Beck argues that people occupy social risk positions and that knowledge allows you to act to mitigate your own risks. Acting becomes a way of virtue signalling, and not mitigating your risk position makes you a “dangerous outsider.” The sorts of horizontal division in society that this creates can be looked at using the analytic construct of moral panics.

## Mods and Rockers Ride Again

The social theory of moral panic was first introduced by Stanley Cohen in his book *Folk Devils and Moral Panics* .^14^ The book focused on public reaction to clashes between two rival youth subculture groups, “mods” and “rockers,” in 1964 over public holiday weekends at beaches in Southern England. On the basis of analysing these clashes and the media and public response to them, Cohen developed a social theory of moral panic comprising five sequential stages:

1. An event, condition, episode or someone is defined as a threat to the values, safety and interest of the wider society.
2. The media then amplifies these apparent threats through inflammatory rhetoric These portrayals appeal to public prejudices, creating villains in need of social control (folk devils) and victims (the moral majority).
3. The publicity surrounding the threat creates a sense of social anxiety leading to a public outpouring of concern.
4. Government then responds to the public outcry and frames the alleged threat as being symptomatic of a wider social malaise that must be addressed.
5. The moral panic and the responses to it transform the regulation of economy and society with the aim of tempering public outrage.

The moral panic Cohen describes appears more characteristic of a form of government in which sovereign power was of central importance; the need for action to address the threat to social order, the way the response to the moral panic transforms the regulation of society and the emphasis on these folk devils needing social control. There is an intention to contain and suppress the transgressors. It is also a moral panic in which the “folk devils” are not seen as active agents beyond their initial transgressions.

Since its original formulation the concept of moral panics has developed with recent approaches subsuming the concept within a wider theory of moral regulation where moral panics are viewed as amplified and volatile expressions of temporary ruptures which occur when the routine processes of moral regulation fail.^15^ This is a formulation of moral panic that is consistent with governmentality, when sovereign power is not needed but routine disciplinary power does not quite seem to be enough invoking a moral panic is sufficient to bring things back under control.

The moral panic that most preoccupies public health today is one about obesity,^16^ often inaccurately characterised as “a disease of affluence.” This moral panic is different to the panic of the 1960s. These differences are best examined using the insights of governmentality and of risk society.

## Obesity and the Body Politic

The World Health Organization (WHO) definition of obesity is, “abnormal or excessive fat accumulation that presents a risk to health.” Obesity is identified within the health service via the use of a widely disseminated measure, body mass index (BMI). BMI is a calculation based on the relationship between height and weight. It will indicate to you, or to your doctor, if you are underweight, healthy weight, overweight, or obese. Obesity then is used as a signifier of a heightened risk of morbidity and of a premature death. It is also an indicator that you are likely to have a greater need for healthcare now and particularly in the future.

Rates of obesity are increasing globally and the ‘obesity epidemic’ is characterized as one of the gravest threats to individual and public health of our times.^17^ Public discourse around obesity displays many of the hallmarks of a moral panic. Those who are labeled obese are demonized in the media as immoral ‘folk devils’ (literally ‘fat devils’) who violate societal values of self-control and who place an avoidable economic burden on national health systems. Pressures to obtain the right body size or shape carries moral connotations which negatively frame those as being overweight or obese as being slothful, lacking self-control and gluttonous. The obesity epidemic is linked with broader political anxieties, poor levels of national fitness and the discomfort of the United Kingdom being seen as “the fat man of Europe” for exampe.^16^

Obesity prevention mass media campaigns (eg, *Strong4Life*  in the United States, *Change4Life*  in the United Kingdom, and *Swap it, don’t stop it*  in Australia) predominantly frame obesity as an issue of personal responsibility – arising from poor individual choices – which can be fixed by individuals taking personal responsibility for weight loss through making better lifestyle choices.

But whereas in Cohen’s example the folk devils were a very small group of people the obese are not! In all but one of the Organisation for Economic Co-operation and Development (OECD) countries plus the US people categorized as obese make up more than a fifth of the adult population (in the United States more than a third) and average adult BMI is higher than that identified as normal (Japan is the exception in both cases). Thus, the average adult is, at least, overweight.^18,19^

In this scenario where folk devils constitute a very significant proportion of the population and where, unlike the mods and rockers, they are not breaking the law, Cohen’s five sequential stages of the manifestation of a moral panic need modification. Rose^8^ has suggested a number of areas that illuminate how problems arise and are responded to in the context of governmentality. Table 1 captures a series of steps that shape the way obesity has become an issue of concern, in so doing it illustrates how this moral panic occurs in the context of governmentality.

**Table 1:** Governmentality and the Moral Panic Around Obesity

**How Problems Arise and Are Responded to**	**Obesity as the Issue and the Obese as the Problem**
Problematization: • How did this problem emerge and what concerns is it in relationship to? • Who defines it as a problem? • How are people with this problem differentiated from those who do not have it?	The issue emerged as non-communicable diseases assumed a priority in public health Obesity was defined as a problem because of a link made between it and some major non-communicable diseases. People are differentiated by BMI – a measure that identifies who is seen as having a healthy weight and who is identified as at risk.
Explanation: • What is the language used to explain? • What is considered to be evidence? What sorts of visibility is conferred?	There are competing discourses: Obesity as an individual responsibility indicating a lack of control, a moral failing. Obesity as a social issue either as a manifestation of a particular modernity or as an outcome of living in an obesogenic environment. Evidence of obesity is linked to an easily arrived at measure (BMI). Obesity is a health and an aesthetic construct – there is a “desirable” body size and shape promoted by the media as well as by health experts.
Technologies: • What tests are used? • What are the techniques of reformation and cure invoked? • How will these be enacted?	Measurement and location on a continuum – with the centre of that continuum being the desired location. Reformation and cure are linked to individuals modifying their behaviour – eating healthy foods and exercising. This ostensibly will be achieved by advice and persuasion, but in practice the mechanism for change relies on seeking to label those resistant to change as morally failing. There is also a discourse that identifies social context, this prompts more sovereign power opportunities – tax/ prohibitions/zoning/not giving planning permissions for fast-food outlets etc.
Authorities: • Who is considered to have expertise? • Who maintains authority in this area?	Psychologists and behavioural economists. Doctors and nurses, with inputs from dieticians. Public health practitioners (town planners and urban geographers) when social dimensions are engaged.
Subjectivities: • What kind are we trying to foster/create?	The health identity: virtuous, wise, moderate. The aspirational aesthetic: slim and therefore attractive, desirable.
Strategies: • What is the governmental aspiration here?	“Prevention of degeneration, eugenic maximisation of the fitness of the race, minimisation of the cost of social maladjustment.”^8^ Also seeking to foster the conformity of the population through the instructional example of the misery of the folk devils.

Abbreviation: BMI, body mass index. Note: Left column adapted from Rose.^8^

## Social Science Weighs In

There is intense critique within the medical sociology and ‘Fat Studies’ literature with regards to the assumed objectivity and neutrality of epidemiological science and its apocalyptic predictions around the so called ‘obesity epidemic.’^5,20-23^ This literature also highlights the creation of new moral panics around ‘classed’ demographics of fatness based on geographical location and social background, around “communities of color”^22^ and especially the moralisation around childhood obesity – which it is argued, serves as a vehicle for the victim – blaming of working-class mothers.^24^ Indeed, it is becoming increasingly clear that the dominant obesity discourse with its emphasis on individual moral responsibility and personal lifestyle modification, ignores biological, social and structural contexts. Between 40%-70% of body weight variance is inherited, with more than 200 genes influencing weight and fat distribution.^25,26^ There are influences that come from endocrine disruptors, from the effects of sleep debt, smoking cessation and from the side effects of prescribed medications.^27^ Obesity also follows social gradients in wealth and inequality^28^ and is influenced by ‘obesogenic’ environmental factors such as poor access to healthy food outlets, a high density of fast food restaurants and a lack of open space for exercise.^29^ There is also the impact of the vested interests of food and drink industries manifest in advertising strategies and in lobbying activity.

LeBesco^30^ claims that the new wave public health approaches to obesity are concomitant with the shift to a neoliberal form of governmentality which normalises certain kinds of bodies under a gaze that constantly keeps deviance under surveillance. Here population statistics function to measure and classify obesity as a type of unhealthy deviance from the norm which is subsequently deemed as a threat to society. Hence citizens are expected to “locate themselves within the BMI scale, to confess being fat and to seek the appropriate bodily discipline (diet and exercise) to avoid becoming an economic burden for society.”^30^ A discourse that associates weight with illness and promotes individual moral responsibility fosters the internalisation of weight-based stigma, engenders negative emotions of guilt, shame and anxiety for those who do not meet socially acceptable weight benchmarks^31^ and attracts other non-weight stigma identities including being morally flawed and being inconsiderate.^3^ Moreover, numerous empirical studies demonstrate that obesity stigma is ineffective in reducing the incidence of obesity as it acts as a stressor which promotes weight gain, has deleterious consequences for mental health (depression anxiety, body dysmorphia), is associated with a range of physical health issues and serves to deepen existing structural inequalities.^31-33^

Moving from sovereign to disciplinary power in public health, changes the repertoire of techniques at its disposal. We can see a shift in emphasis from large scale public works, via the use of the law to the now fashionable idea of nudging people to change.^34^ Nudge is a behavioural economic approach, one of a number that have become increasingly influential in public health as tools for influencing behavior change and encouraging ‘better’ choices. The approach is based on the somewhat oxymoronic notion of “libertarian paternalism” – a new branch of neoliberal governmentality.^35^ It assumes that policy-makers should not deny people options but that they should consider manipulating the choice architecture to promote “better” choices. Examples include: provision of information – calorie counts on menus, and changes to the default option – salad rather than chips. Nudge both individualises understandings of choice in relation to health and fosters paternalism in pre-setting the choice architecture. Its use augments and in some cases replaces a range of other techniques:

- *Hugs* : financial incentives such as vouchers in exchange for healthy behavior.
- *Shoves* : measures that restrict choice, like increasing the price of cigarettes or limiting takeaways and fast food outlets near schools.
- *Smacks* : Bans, such as the restriction on smoking in public places.

## Disciplinary Power Does not Eliminate Agency

While Cohen’s mods and rockers may have manifest personal and group agency as they clashed on the beaches the ensuing moral panic, and the use of sovereign power, did not allow for their continuing to have an active role. But disciplinary power generates resistance, marginalised groups seek to oppose dominant narratives and to offer alternatives. These efforts are opposed and their resonance is diminished by their being characterised as “special cases” but, over time, solidarities can emerge and counter narratives can be offered and sustained.^7^ This is a process that is evident in relation to obesity and is summarised in Table 2.

**Table 2:** Where There Is Power, There Is Resistance

**Elements That Can Be Challenged in the Exercise of Disciplinary Power**	**The Discursive Resistance of TheMarginalised**
Problematization/ technologies: • Against the dominant discourse • Against prevailing classifications/measurement	Against the discursive association of body size as a proxy for health. Against diet/exercise “choices” as explanations for trends in obesity. Critical of the “artifactually constructed” idea that you use BMI to measure adiposity, you link excess deaths with high BMI and that you define high, normal and low according to the characteristic bell curve of BMI scores.^36^
Explanations: • Challenging “accepted” evidence • Deconstructing motivations	Questioning the assumed connections of body size and health and the effectiveness of strategies to reduce obesity that target behaviour change in those identified as most at risk of adverse health impacts. Ask “who benefits from the prevailing obesity discourse” and “who benefits from the creation and maintenance of an obesogenic environment?” The former privileges the profession of medicine and the latter requires scrutinising the profit motive in the producers and distributors of “fast food” and other high calorie/high sugar food and drink.
Authorities/subjectivities	Critical of using medical terms to classify body size. In the same way as other oppressed groups have questioned terminologies defined by authorities external to the affected group (eg, homosexual) so obesity, it is argued, can be replaced by “fat,” see the Fat Underground, the Fat Liberation Manifesto and including the scholarship presented in the journal “Fat Studies.”^37^ The argument is that both a critical examination of societal attitudes about body weight and appearance and advocating for equality for all people irrespective of body size are needed.
Strategies	Opposing the use of stigma as a deliberate policy to encourage weight loss. Creating solidarities of the marginalised to challenge the dominant discourse instead of isolating and individualising them.

Abbreviation: BMI, body mass index.

The invocation of disciplinary power also relies on the communication of messages, but there is a paradox in that those who frame the message are not always in control of its communication. The media does not just communicate medical and scientific messages it also uses these messages in ways that serve the interests of the media, for example exaggerating the dramatic and alarmist elements of academic reports in ways that are believed to best satisfy its readers/viewers.^38^

## Putting Governmentality, Individualised Public Health and Moral Panics Together

A consequence of the increased emphasis on individual responsibility for ill health means that neither state, civil society or private sector institutions are held responsible for health problems. Blaming individuals and groups for the health needs they manifest leads to a focus on disciplinary power and, in so doing, ignores underlying biological and structural causes and puts undue and counter-productive pressure on the vulnerable. This is not to argue a fatalist approach where individuals have no agency or self-determination in relation to maintaining their health. But creating folk devils and mobilising moral panics is a risky tactic, it is something that might be invoked for other issues where there is a reluctance to use sovereign power and where governmentality is not quite enough. Hier^39^ has talked of “panic as regulation” approaches. These sorts of issues are vulnerable to appropriation by right wing populism. Here health fears are politicised, simplified, and made spectacular, for example over immigrants accessing healthcare, or fears over malign experts in pocket to big Pharma promoting dangerous vaccination on a vulnerable populous.^40-42^ Fuelling moral panics and creating folk devils are blunt public health tools with the potential for consequences that bludgeon the vulnerable.

## Ethical issues

Not applicable.

## Competing interests

Authors declare that they have no competing interests.

## Authors’ contributions

Both authors contributed equally to the writing of this article.

## Authors’ affiliations

^1^Health Services Management Centre, University of Birmingham, Birmingham, UK. ^2^Faculty of Health Studies, University of Bradford, Bradford, UK.
